# Transcriptome Analysis Reveals Antioxidant Defense Mechanisms in the Silkworm *Bombyx mori* after Exposure to Lead

**DOI:** 10.3390/ani14121822

**Published:** 2024-06-19

**Authors:** Yang Ye, Yan-Xia Shi, Qi Jiang, Ye Jin, Fan-Xing Chen, Wen-Hui Tang, Qin Peng, Qiu-Ning Liu, Bo-Ping Tang, Jia-Lian Wang

**Affiliations:** 1 Jiangsu Key Laboratory for Bioresources of Saline Soils, Jiangsu Synthetic Innovation Center for Coastal Bio-Agriculture, Jiangsu Provincial Key Laboratory of Coastal Wetland Bioresources and Environmental Protection, School of Wetlands, Yancheng Teachers University, Yancheng 224007, China; 2 College of Biotechnology and Pharmaceutical Engineering, Nanjing University of Technology, Nanjing 210009, China; 3 College of Marine and Biological Engineering, Yancheng Teachers University, Yancheng 224007, China

**Keywords:** *Bombyx mori*, midgut, lead, transcriptome, gene expression, antioxidant defense mechanisms

## Abstract

**Simple Summary:**

Lead is a major source of heavy metal pollution, which can harm both the environment and people’s health. High levels of Pb can slow down the growth and development of insects and may even cause them to die by damaging their cells. The aim of this study was to explore gene expression in the midgut of *Bombyx mori* when exposed to lead (Pb). This study gives us new insights into how insects protect themselves from Pb challenge and helps us understand the complex ways insects get rid of harmful substances at a deeper level.

**Abstract:**

Lead (Pb) is a major source of heavy metal contamination, and poses a threat to biodiversity and human health. Elevated levels of Pb can hinder insect growth and development, leading to apoptosis via mechanisms like oxidative damage. The midgut of silkworms is the main organ exposed to heavy metals. As an economically important lepidopteran model insect in China, heavy metal-induced stress on silkworms causes considerable losses in sericulture, thereby causing substantial economic damage. This study aimed to investigate Pb-induced detoxification-related genes in the midgut of silkworms using high-throughput sequencing methods to achieve a deeper comprehension of the genes’ reactions to lead exposure. This study identified 11,567 unigenes and 14,978 transcripts. A total of 1265 differentially expressed genes (DEGs) were screened, comprising 907 upregulated and 358 downregulated genes. Subsequently, Gene Ontology (GO) classification analysis revealed that the 1265 DEGs were distributed across biological processes, cellular components, and molecular functions. This suggests that the silkworm midgut may affect various organelle functions and biological processes, providing crucial clues for further exploration of DEG function. Additionally, the expression levels of 12 selected detoxification-related DEGs were validated using qRT-PCR, which confirmed the reliability of the RNA-seq results. This study not only provides new insights into the detoxification defense mechanisms of silkworms after Pb exposure, but also establishes a valuable foundation for further investigation into the molecular detoxification mechanisms in silkworms.

## 1. Introduction

With the rapid development of global industrialization, heavy metal pollution caused by activities such as mining, metal processing and smelting, chemical manufacturing, and factory emissions has become a notable environmental concern in numerous regions across the globe [1,2]. The primary heavy metals of concern comprise chromium (Cr), copper (Cu), zinc (Zn), cadmium (Cd), mercury (Hg), and lead (Pb) [3].

The accumulation of excessive heavy metal ions in soil causes extensive damage to the ecological environment, significantly reducing soil productivity and occasionally leading to degradation [4]. Moreover, heavy metal ions pose a threat to plants, animals, and humans through ecological cycles [5]. In the early 20th century and from the 1950s to the 1970s, Japan witnessed infamous incidents of metal contamination, such as Itai-itai disease and Minamata disease, leading to the deaths of thousands of individuals [6,7]. In 2016, a water contamination incident in Flint, Michigan, USA, resulted in hundreds of local children being detected with elevated blood Pb (PbB) levels above normal ranges [8]. Pb, which has been used by humans since 4000 BCE, was one of the earliest metals discovered by humans [9]. Ancient civilizations, such as the Romans, extensively used Pb for constructing water pipes owing to the advanced engineering technology available at the time. However, research progress has led to the discovery that this application might pose public health concerns because Pb breakdown in water can be harmful to human health [10,11]. In the Balkan Peninsula region, the development of mineral resources has led to significant Pb pollution, recorded at approximately 600 BCE. Human activity-induced Pb content gradually increased, reaching its peak at approximately 1620 BCE [11]. During the Industrial Revolution in Europe from the 19th to 20th centuries, Pb was commonly used in industries like batteries, printing, coatings, and pipelines, leading to significant pollution. For example, the deposition of atmospheric Pb in peat bogs in rural parts of western and central Switzerland increased significantly from 40 to 80 times between 1880 and 1920, and from 80 to 100 times between 1960 and 1980, increasing risks to both the local human population and the ecological environment [12]. Despite its widespread use in various industries, including battery production, construction, automobile manufacturing, and military applications, its use has been restricted to certain applications, such as Pb pipes and Pb-based paints, owing to environmental and health concerns [13,14]. The evolving awareness and understanding of the harmful impacts of Pb on human health and the environment have prompted more cautious use and management practices over time.

The silkworm (*Bombyx mori*), belonging to the order Lepidoptera and family Bombycidae, is a unique silk-producing insect of significant cultural and economic importance in China. Since the Xia Dynasty, China has been domesticating silkworms by selectively breeding them from mulberry tree pests and developing sericulture techniques. This tradition has persisted to the present day, continually contributing to economic benefits. However, prolonged domestication has resulted in silkworms gradually losing their resistance to drugs and pathogenic microorganisms [15,16,17]. Heavy metal ions and other harmful substances absorbed by mulberry trees accumulate in their leaves. Silkworms feed on these leaves, which negatively affects their growth, development, and silk production, which in turn adversely impacts the silk industry [18,19]. Therefore, investigating the toxic effects of heavy metals on silkworms is imperative.

The sensitivity of silkworms to heavy metals, drugs, and pesticides has made them ideal model organisms for assessing health and safety as well as studying environmental pollution mechanisms [16,20,21]. Therefore, in-depth research on the toxic effects of harmful substances such as heavy metals on silkworms is essential. This will enable the implementation of appropriate protective measures, ensuring the sustainable development of the silk industry while safeguarding the ecological environment and human health. Several studies have been conducted to reduce the damage caused by Pb by better understanding its adverse effects and toxic mechanisms. For instance, research on the housefly (*Musca domestica*) has shown that Pb stress causes changes in the morphology of its blood cells, with an increase in the original blood cells and a decrease in phagocytic cells, leading to impaired phagocytic function and weakening of the organism’s repair capacity [22]. Similarly, studies on tobacco cutworm (*Spodoptera litura*) have shown that Pb and Zn stress increases the gaps between midgut cells, disrupts mitochondrial structure, and ruptures the nuclear membrane [23,24]. In contrast, the accumulation of Cd and Pb in the intestines of mice (*Mus musculus*) was relatively low. However, Cd exhibits significant genotoxic effects in both the upper and lower gastrointestinal tract [25]. Studies in the soil-mulberry silkworm-silkworm food chain have shown that Pb ions can accumulate in various parts of silkworms, such as larvae, feces, and silk, via a translocation process [26]. However, many uncertainties remain regarding the molecular toxicology of Pb ions, particularly the molecular detoxification mechanisms of animals and the molecular-level toxic effects of Pb ions. Studies on invertebrates, particularly insects, are limited, and there are still many unknown areas that require further exploration.

RNA sequencing, also known as RNA-seq, is a transcriptome analysis technique that utilizes deep sequencing technology to produce transcriptome profiles. When compared with alternative methods, RNA-seq is a reliable method for accurately quantifying the levels of specific transcripts. Additionally, RNA-seq contributes to improving our understanding of the host’s genetic responses to various chemical elements and their underlying molecular mechanisms [27]. Pb, a toxic heavy metal, can cause damage to numerous organs in the body [28]. In this study, the silkworm was chosen as a model organism to investigate the toxicity mechanism of Pb [29]. The larval midgut, responsible for digesting mulberry leaves and absorbing nutrients, was selected as the target organ for the toxicological experiments. Heavy metal ions enter the organism through this organ, which is also considered an important immunological barrier against exogenous factors and pathogens [30,31]. A comprehensive transcriptome analysis of the larval midgut was conducted to better understand the toxicity of Pb and the mechanisms of the silkworm response to Pb. One group was fed normal mulberry leaves, whereas the other group was fed mulberry leaves soaked in a solution of C_4_H_6_O_4_Pb·3(H2O). The toxic effects of Pb were analyzed by identifying differentially expressed genes (DEGs).

## 2. Materials and Methods

### 2.1. Silkworm Strain and Lead Exposure

The hybrid silkworm variety “Jingsong × Haoyue” was selected as the experimental model. The rearing conditions for the silkworm larvae were as follows: a temperature of 27 °C, a humidity level of 70% to 75%, a light cycle of 12 h light and 12 h darkness, and fresh mulberry leaves were provided as food three times a day.

Lead acetate (C_4_H_6_O_4_Pb·3(H_2_O)) purchased from Fuchen (Tianjin) Chemical Reagent Co., Ltd., with a purity of ≥99.5%. Dissolve 20 mg of lead acetate raw material in 1mL of double-distilled water to make the stock solution, then dilute the stock solution with double-distilled water to prepare working solutions of 0.2 mg/L. Immerse mulberry leaves in 500 mL of imidacloprid working solution for 1 minute, air dry, and use to feed 5th instar larvae starting from the third day, three times a day, until cocooning. Each experimental group consists of 20 larvae with three biological replicates. The control group’s mulberry leaves were treated with double-distilled water (ddH_2_O).

### 2.2. Determination of Enzyme Activity

After being exposed to trace amounts of lead (Pb), the detoxifying enzyme activity was evaluated in both the control (CK) and lead-exposed (Pb) groups. The P450 enzyme activity was determined using a cytochrome P450 assay kit from Grace Biotechnology Ltd., Suzhou, Jiangsu, China, following the company’s instructions. Additionally, the activities of the GST and CarE enzymes were assessed using kits from Nanjing Jiancheng Bioengineering Institute, Nanjing, Jiangsu, China, and the manufacturer’s guidelines were followed for these measurements.

### 2.3. Midgut Collection and RNA Extraction

At the 48-h mark post lead (Pb) exposure, a random selection of 30 fifth instar larvae was made. The midguts were swiftly extracted, chilled on ice, and dissected to eliminate their contents. They were then washed with a cold solution of phosphate-buffered saline (pH 7.4). Isolation of total RNA from these midgut tissues was carried out with TRIzol reagent (Invitrogen, Carlsbad, CA, USA), along with the use of chloroform, isopropanol, 75% ethanol, and DEPC-treated water, all in accordance with the manufacturer’s guidelines. Subsequent to the extraction process, the RNA was checked for any signs of degradation and contamination through a 1% agarose gel analysis. The RNA’s purity, concentration, and integrity were determined using the NanoDrop (Impen, Westlake Village, CA, USA), Qubit 2.0 (Life Technologies, Carlsbad, CA, USA), and Agilent 2100 (Agilent Technologies, Santa Clara, CA, USA) instruments, respectively.

### 2.4. Library Construction and High-Throughput Sequencing

A cDNA library was constructed using the Seq RNA Sample Prep Kit (Illumina, San Diego, CA, USA). mRNA was enriched using magnetic beads coated with oligo(dT). Subsequently, the mRNA was fragmented using fragmentation buffer. Single-stranded cDNA was synthesized using mRNA as a template using reverse transcriptase and random hexamers. Subsequently, second-strand synthesis was carried out to generate a stable double-stranded structure. These double-stranded cDNA fragments were then end repaired, and an ‘A’ base was inserted at the 3′ end. Sequencing adapters were ligated. PCR amplification was performed and the resulting amplicons were purified using AMPure XP beads to obtain the final cDNA library. Transcriptome sequencing of the cDNA libraries from the ck and Pb-exposed groups was performed using an Illumina HiSeq 2000 platform.

### 2.5. De Novo Assembly and Functional Annotation

Illumina HiSeq sequencing generated raw data (raw reads) and high-quality clean data were obtained after quality control processes, such as removal of low-quality sequences and adapters. The obtained clean reads were assembled de novo using Trinity software v2.15.1 on the reference transcriptome. TransDecoder software v5.3.0 was used to predict the coding region sequences and the corresponding amino acid sequences of the unigenes. To obtain comprehensive gene functional information, the unigene sequences were compared with the NCBI non-redundant protein/nucleotide sequences (Nr) [32,33], Swiss-Prot (a manually annotated and reviewed protein sequence database) [34], Protein family (Pfam) [35], Clusters of Orthologous Groups of proteins (eggNOG) [36], Gene Ontology (GO), and Kyoto Encyclopedia of Genes and Genomes (KEGG) [37] databases using the BLAST software v2.9.0. This comparison facilitated the annotation of the unigenes with relevant information.

### 2.6. Differential Gene Expression and Gene Enrichment Analysis

Bowtie program [38] was used to align the reads obtained from sequencing various samples with the unigene library. Expression levels were estimated based on the alignment results combined with RSEM software v1.3.3 [39]. Gene expression calculations used fragments per kilobase per million (FPKM) values [40], which can be directly compared between different samples to assess gene expression differences. DEGSeq software v1.30.0 [41] was then used to identify genes with differential expression across sample groups. The filtering process involved the use of IDEG6 software for chi-square tests, and the obtained *p*-values were corrected using multiple hypothesis testing (false discovery ratio [FDR]). Genes with FDR < 0.01 and a fold change in FPKM ratio of 2 or more (|Fold Change| ≥ 2) between samples were considered as differentially expressed genes.

Subsequently, GOSeq R [42] and KOBAS [43] were used for GO functional enrichment and KEGG pathway enrichment analyses of DEGs to further investigate their potential roles. Gene enrichment analysis utilizes biological information databases and statistical tools to enrich specific genes in known functional biological pathways or modules, thereby providing a better understanding of gene function from a biological perspective.

### 2.7. Quantitative Reverse-Transcription PCR (qRT-PCR) Analysis

For the comparison between the 48 h ck and 48 h Pb-exposed groups, 12 DEGs were randomly selected for qRT-PCR analysis. Primers were designed using the Primer Premier 5 software (Table S1). Each sample contained three biological replicates. RNA was extracted and reverse-transcribed into cDNA using the PrimeScript^®^ RT reagent kit with gDNA Eraser (Vazyme, Nanjing, China) kit, following the manufacturer’s instructions. β-Actin was used as the internal reference. The SYBR^®^ Premix Ex Taq^TM^ II kit (TaKaRa, Kusatsu, Japan) was used and quantitative fluorescence detection was performed using the Applied Biosystems detection system (Bio-Rad, Hercules, CA, USA). Each experiment was performed in triplicate. Relative gene expression was determined using the (2^–∆∆Ct^) Ct method [44]. Single-factor analysis of variance was conducted using SPSS 20.0 software, and *p* < 0.05 indicated statistically significant differences.

## 3. Results and Discussion

### 3.1. Determination of Enzyme Activity

**Figure 1:**
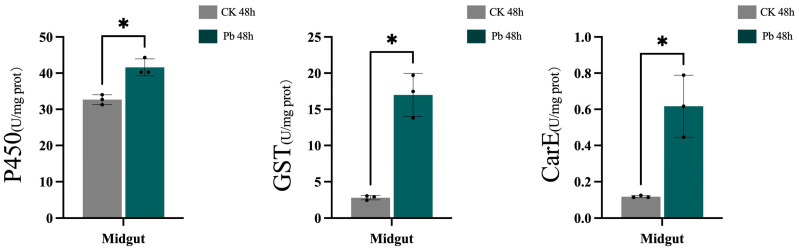
The enzyme activity indicators of P450, GST, and CarE in the midgut of the 48 h control group and the 48 h Pb treatment group. The activity of detoxifying enzymes under lead (Pb) stress was demonstrated. The values were significantly different to the control at the same time point when marked with asterisks (* *p* < 0.05).

Given its current prevalence and harmfulness, lead is considered the second most hazardous environmental toxin. As shown in Figure 1, there was a general increase in the activity of the P450 detoxification enzyme. The activity of the GST enzyme showed a significant increase. Furthermore, the activity of the CarE enzyme was markedly enhanced compared with the control group. The alterations observed in these biochemical markers suggest that exposure to lead (Pb) triggered the detoxification mechanisms within the silkworm, *B. mori*. Several studies have shown toxic effects of lead on different insects such as honeybees (*A. mellifera*) [45], the tobacco cutworm (*S. litura*) [46], and the fruit fly (*Drosophila melanogaster*) [47]. The results show that the midgut of insects has a beneficial detoxification reaction that boosts their ability to withstand exposure to lead.

### 3.2. De Novo Assembly of the Transcriptome

**Figure 2:**
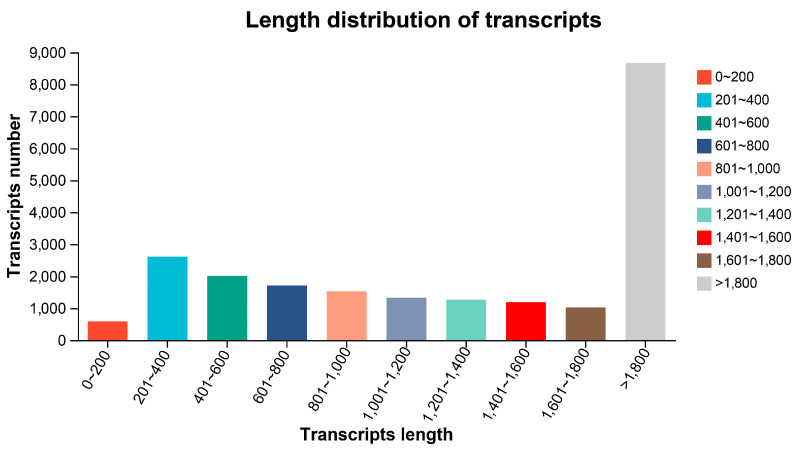
Length distribution of transcripts. The x-axis represents the range of transcript lengths, while the y-axis represents the number of transcripts within each length range.

The ck and Pb-exposed groups yielded 54,308,482 and 43,522,228 raw reads, respectively. After filtering out low-quality reads, short sequences, and low-complexity sequences, 51,994,280 and 41,385,924 clean reads were obtained from the ck and Pb-exposed groups, respectively. The number of clean bases for both groups was 7,679,161,985 and 6,128,133,619, respectively. The Q30 values for the two groups were 93.72% and 93.78%, respectively. The G+C content was 51.78% and 50.92% in the ck and Pb-exposed groups, respectively. Trinity software detected 14,723 transcripts. A total of 11,320 unigenes were identified. Additionally, using the transcripts assembled by Trinity as a reference sequence (ref), the aligned clean reads were 42,649,323 (82.03%, ck) and 34,592,406 (83.58%, Pb-exposed) (Table S2). The assembly results showed that 5203 transcripts were within the range of 0–600 bp, 4564 transcripts were within the range of 600–1200 bp, 3474 transcripts were within the range of 1200–1800 bp, and 8666 transcripts were >1800 bp (Figure 2, Table S3). These findings indicate data of high quality, reliable unigenes, and suitability for annotation analyses.

We used StringTie software v2.2.0 to assemble each sample individually and then merged them together. Subsequently, we conducted statistical analysis and visualization on the assembled results.

### 3.3. Functional Annotation of Unigenes

To gain a more comprehensive understanding of the molecular functions of these genes, unigenes were compared using six different databases. The number of unigenes that were successfully matched with each database was as follows: NR, 16,195 (91.55%); Swiss-Prot, 10,306 (58.26%); Pfam, 10,950 (61.9%); EggNOG, 15,253 (86.22%); GO, 12,460 (70.44%); and KEGG, 8476 (47.91%) (Table S4).

### 3.4. Analysis and Identification of Differentially Expressed Genes (DEGs)

**Figure 3:**
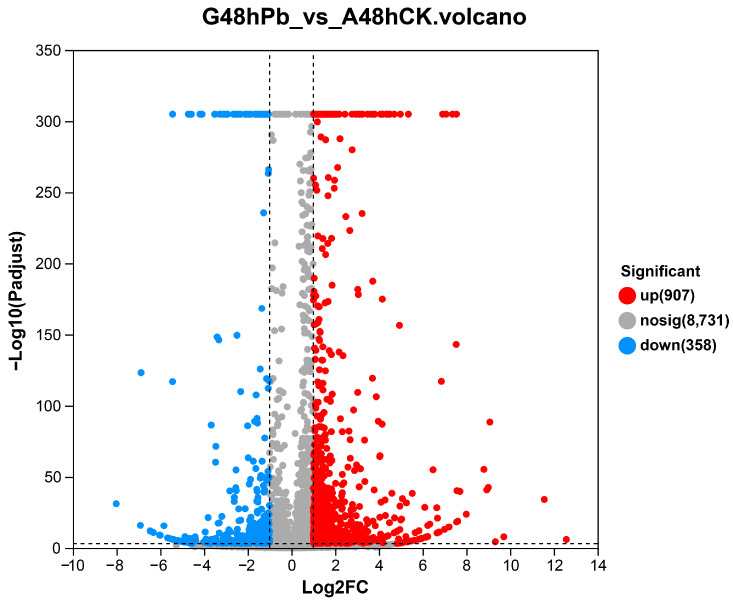
Volcano plot of DEGs. The x-axis represents the fold-change in gene expression between the control group (ck) and the group exposed to lead (Pb), and the y-axis signifies the statistical significance of this differential expression. On this graph, red dots denote DEGs that are significantly upregulated, blue dots indicate DEGs that are significantly downregulated, and gray dots correspond to genes with expression changes that do not reach statistical significance (where the q-value is less than 0.005 and the absolute value of log2 fold-change is greater than 1).

DEG-seq was used to analyze all unigenes, with a threshold set at P_adjust_ < 0.001 and log2 (fold-change) ≥ 1 [48]. A total of 1265 significant DEGs were identified between the Pb-exposed and ck groups, comprising 907 upregulated and 358 downregulated genes (Figure 3). In the volcano plot, red, blue, and gray dots represent significantly upregulated unigenes, significantly downregulated unigenes, and unigenes with no significant differential expression, respectively. The extent of expression change in upregulated differentially expressed genes (DEGs) was found to be less pronounced than that in downregulated DEGs (Figure 2). Our results indicate that this particular gene expression profile could be associated with exposure to Pb.

### 3.5. GO Functional Classification Analysis of the DEGs

**Figure 4:**
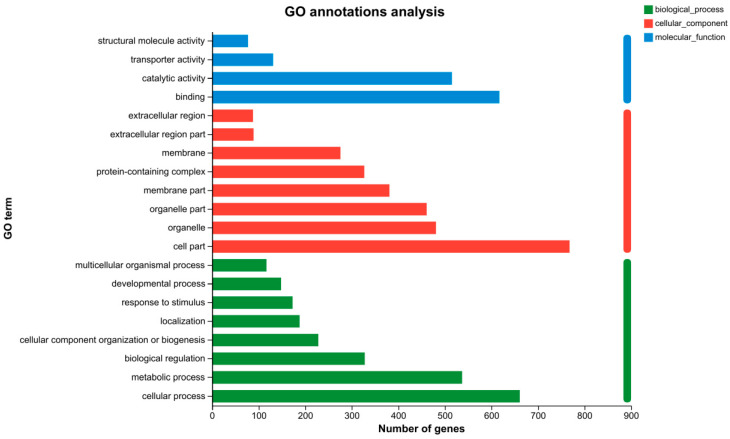
GO classification of transcriptomic DEGs in *Bombyx mori* midguts. Each annotated sequence is assigned at least one of the following GO terms: biological process, cell component, or molecular function.

GO classification is a comprehensive gene function classification system that comprises three ontologies: biological process (BP), cellular component (CC), and molecular function (MF). Functional analysis of DEGs was conducted using the Blast2GO software and the GO database. In this study, 1,265 DEGs were categorized into three primary categories. Figure 4 shows the top 22, 14, and 10 enriched GO terms in the BP, CC, and MF categories, respectively. In the BP category, “cellular process” (661 DEGs), “metabolic process” (537 DEGs), “biological regulation” (328 DEGs), and “cellular component organization or biogenesis” (228 DEGs) were the most enriched subcategories. CC included “cell part” (768 DEGs), “organelle” (481 DEGs), “organelle part” (461 DEGs), “membrane part” (381 DEGs), and “protein-containing complex” (327 DEGs). In MF, “binding” (617 DEGs), “catalytic activity” (515 DEGs), “transporter activity” (131 DEGs), and “structural molecule activity” (77 DEGs) were predominant. These findings suggested that Pb stimulation in the silkworm midgut may involve various cellular functions and biological processes, providing valuable resources for investigating the potential functions of DEGs.

### 3.6. KEGG Pathway Analysis of DEGs

**Figure 5:**
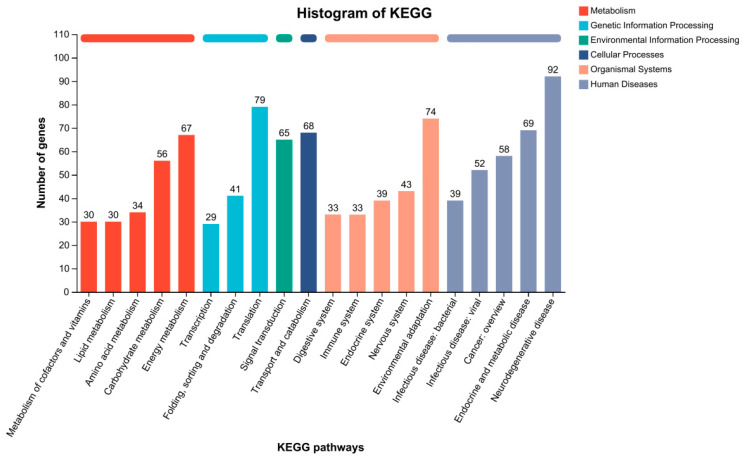
KEGG classification map of DEGs. The x-axis indicates both the count of genes associated with a specific pathway and the ratio of these genes to the overall number of genes that have been annotated. Meanwhile, the y-axis displays the names of the respective KEGG metabolic pathways.

KEGG is a bioinformatics database used for gene function analysis, providing valuable categorization for understanding the complex biological functions of genes [43]. Therefore, we conducted KEGG pathway annotation to investigate the biological functions and metabolic pathways of these DEGs between the ck and Pb-exposed groups (Figure 5). The KOBAS software was used to map all DEGs to terms in the KEGG database and allocate them to six KEGG biochemical pathways, including “Metabolism”, “Genetic Information Processing”, “Environmental Information Processing”, “Cellular Processes”, “Organismal Systems”, and “Human Diseases.” The results revealed that DEGs were most enriched in “Translation” (79 DEGs), followed by “Environmental adaptation” (74 DEGs), “Transport and catabolism” (68 DEGs), and “Energy metabolism” (67 DEGs). This suggests that Pb may affect the digestive and metabolic systems of silkworms.

**Figure 6:**
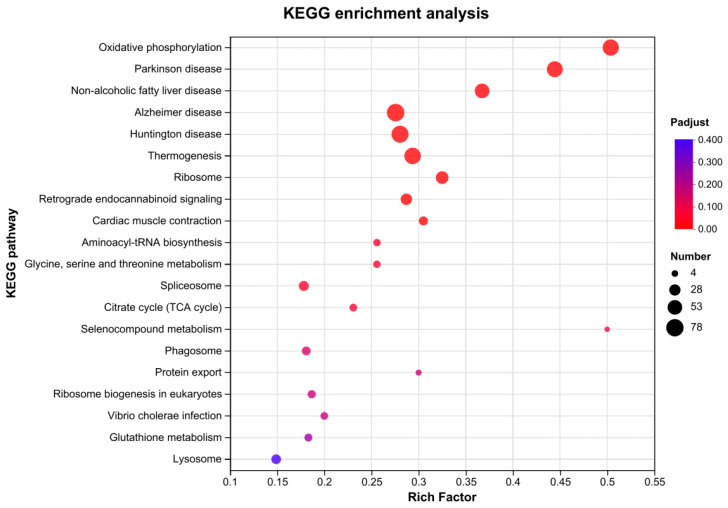
Map of rich hubs in the KEGG pathway of DEGs. The x-axis represents the enrichment factor and the y-axis indicates the pathway name.

Among all the genes with KEGG annotations, 805 DEGs were assigned to 297 KEGG pathways. A scatter plot of the top 20 enriched pathways with a *p*-value ≤ 0.05 is shown in Figure 6. These enriched pathways were broadly categorized into three functional categories: “Transport and catabolism”, “Carbohydrate metabolism”, and “Digestive system”. The pathways related to “Transport and catabolism” include “Lysosome” and “Phagosome”. “Carbohydrate metabolism-related pathways” include “Oxidative phosphorylation”. Pathways related to the “Digestive system” include “Thermogenesis”, “Retrograde endocannabinoid signaling”, and “Cardiac muscle contraction” (Figure 6). Among these pathways, lysosomes were of particular interest because they are the primary degradative organelles in most eukaryotic cells [49]. They possess an acidic lumen, consisting of approximately 60 acidic hydrolases that function as a critical intracellular digestive system and play a crucial role in maintaining cellular homeostasis [50]. Therefore, Pb may affect individual development by influencing the digestion of midgut nutrition. In recent years, lysosomes have been recognized as central hubs for cellular metabolism, with significant cell-signaling regulators located on the lysosomal surface [51].

### 3.7. EggNOG Functional Classification of DEGs

EggNOG is a publicly accessible database containing homologous groups of proteins at different classification levels, each with integrated and summarized functional annotations [52]. EggNOG annotation reveals that “Posttranslational modification, protein turnover, chaperones (O)” (136 instances) have the highest representation, indicating the significant biological role of this functional category in the studied organism. It involves processes such as protein post-translational modifications, protein degradation and folding, as well as molecular chaperones. Therefore, this result suggests that these biological processes may be highly active and important in the studied organism. Following this, “Translation, ribosomal structure and biogenesis (J)” (109 instances) is the second most represented category, which encompasses functions related to RNA translation, ribosomal structure, and biogenesis. This indicates that protein synthesis is a crucial component of organismal survival and function, with these genes likely playing important roles in maintaining cellular function and life processes.

**Figure 7:**
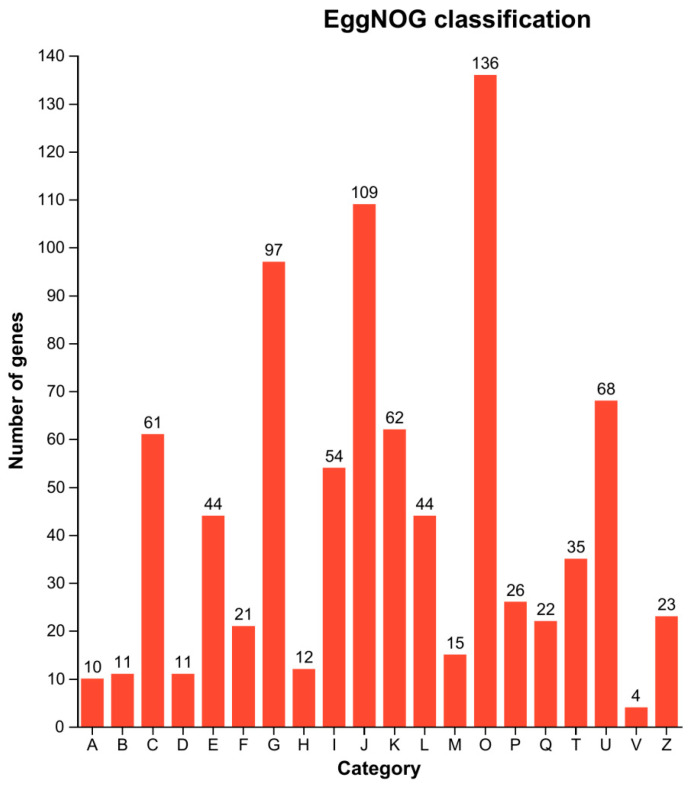
COG annotated classification statistical map of DEGs. The x-axis represents the COG functional annotation class and the y-axis represents the gene number. A for RNA processing and modification, B for chromatin structure and dynamics, C for energy production and conversion, D encompasses cell cycle control, cell division, and chromosome partitioning. Additionally, E pertains to amino acid transport and metabolism, F to nucleotide transport and metabolism, G to carbohydrate transport and metabolism, H to coenzyme transport and metabolism, I to lipid transport and metabolism, and J to translation, ribosomal structure, and biogenesis.

Additionally, “Carbohydrate transport and metabolism (G)” (97 instances) is represented, involving functions related to carbohydrate transportation, degradation, and utilization. This suggests the importance of carbohydrates in metabolism, with these genes likely playing key roles in maintaining energy balance and life processes. Furthermore, four DEGs are associated with “Defense mechanisms (V)”. This suggests that these genes may play important roles in the organism’s defense against external pressures, pathogens, or other harmful factors. These differentially expressed genes may participate in biological processes such as immune responses, antioxidant stress responses, and toxin metabolism, indicating their potential regulatory roles in the organism’s adaptability and survival capabilities (Figure 7).

### 3.8. Validation of Differentially Expressed Genes Using qRT-PCR

**Figure 8:**
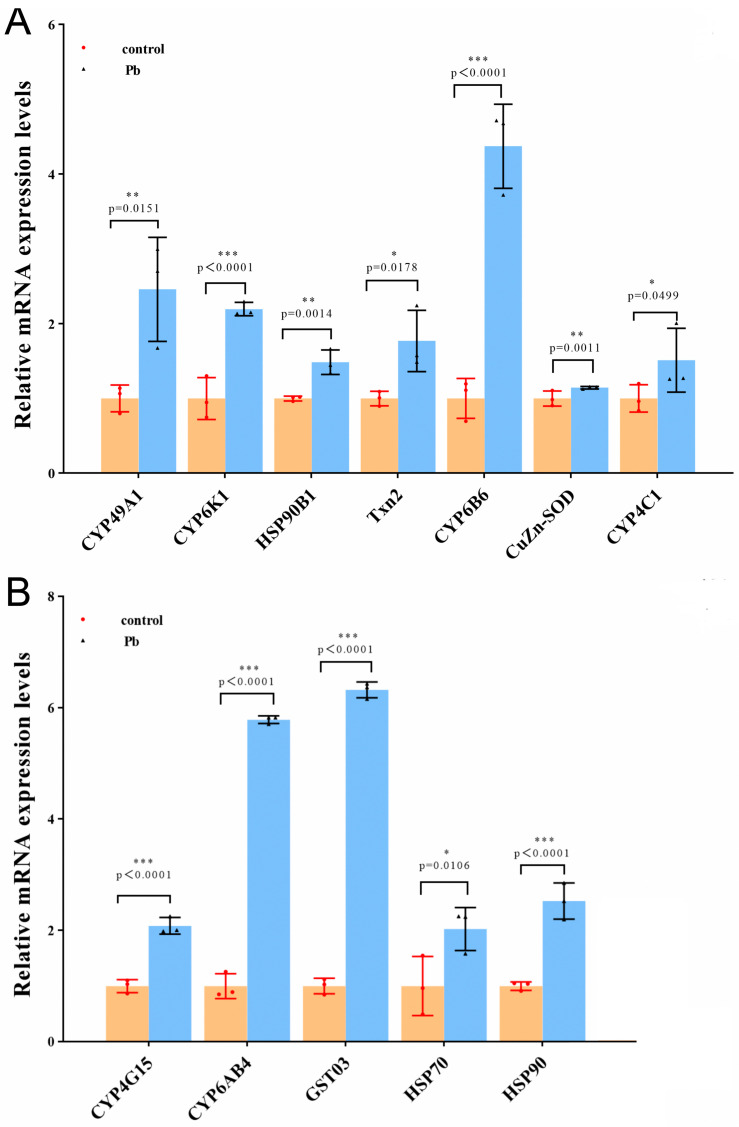
Relative mRNA expression levels of DEGs detected by qRT-PCR. (**A**,**B**) The expression level of each gene in the ck group was normalized. The values were significantly different to the control at the same time point when marked with asterisks (* *p* < 0.05, ** *p* < 0.01,*** *p* < 0.001).

To further validate the reliability of the RNA-seq data, we randomly selected 12 DEGs related to detoxification and antioxidant defense, including CYP49A1, CYP6K1, CYP6B6, CYP4C1, CYP4G15, CYP6AB4, GST03, Txn2, CuZn-SOD, HSP70, HSP90, and HSP90B1. The expression levels of these genes were detected using qRT-PCR. The results showed that, compared to the ck group, the expression trends of the genes in the Pb-exposed group were consistent with the transcriptome expression analysis, confirming the reliability of the RNA sequencing results (Figure 8).

While the midgut of silkworms is primarily responsible for food digestion and nutrient absorption, it also metabolizes exogenous toxins, such as toxic plant secondary metabolites and insecticides [53]. Studies have revealed the presence of various detoxification enzymes in the midgut that play vital roles in various biological processes. Cytochrome P450 enzymes (CYP), which are widely distributed in insect tissues, particularly in the midgut, function as Phase I detoxification enzymes that directly interact with numerous endogenous and exogenous substrates to reduce their toxicity efficiently [54,55]. Guo et al. demonstrated the significant role of CYP subfamily genes in the insecticide metabolism and resistance of locusts, addressing the functional and regulatory mechanisms of the CYP6F subfamily genes in locusts [56]. Glutathione-S-transferases (GSTs) are key detoxification enzymes that catalyze the reduction of exogenous and Phase II endogenous substances using glutathione [57]. Montella et al. [58] highlighted the crucial role of GSTs and glutathione (GSH) in the detoxification of peroxides and oxidized DNA bases, providing a vital protective mechanism. These detoxification enzymes collaborate in the midgut to ensure efficient processing of harmful substances from the environment, thereby maintaining the physiological balance and adaptability of insects. The thioredoxin (TXN) system, an NADPH+ H + /FAD redox effector, promotes cellular survival by maintaining the internal balance, bioenergy, detoxification networks, and preventing oxidative stress-related diseases. Research by Jun Lu et al. [59] suggested that antioxidant functions include DNA and protein repair, reduction of ribonucleotide reductase, methionine sulfoxide reductase, and regulatory enzymes, as well as modulation of numerous oxidative stress-sensitive transcription factors. Superoxide dismutase (SOD) is a ubiquitous enzyme family that catalyzes the dismutation of superoxide anions effectively. A study by Adesina [60] reported that SOD activity in insects treated with herbal insecticide formulations increased with the concentration and exposure time. Increased concentrations of active components in all treated samples stimulated SOD production, resulting in higher dismutation of superoxide anions (O^2−^). Heat shock proteins (HSP) are essential molecular chaperones that play a significant role in insect response to stress stimuli. Wang et al. [61] reported that HSPs confer thermal or cold resistance to whiteflies (*Bemisia tabaci*), protecting them from the adverse effects of temperature conditions, emphasizing the molecular evolutionary characteristics and response mechanisms of HSP genes in whiteflies under temperature stress. Detoxification enzymes in silkworms include P450, GST, and CarE [31]. As many as 17 P450 enzymes are reported to be highly expressed in the intestine of silkworms, including CYP4, CYP6, and CYP9, indicating their significant role in metabolizing metal toxins and under metal stress [62]. Previous studies have shown upregulation of P450 gene expression in the intestine of silkworms exposed to fenvalerate, indicating their importance in fenvalerate metabolism. In this study, after 48 hours of lead exposure, P450 enzyme activity significantly increased, and the transcription levels of genes such as CYP49A1, CYP6K1, CYP6B6, CYP4C1, CYP4G15, CYP6AB4, GST03, Txn2, CuZn-SOD, HSP70, HSP90, and HSP90B1 increased upon exposure to lead for 48 hours. These results are consistent with observations articles, who found increased expression of P450 genes in tobacco cutworms exposed to lead [63,64]. Additionally, the GST03 gene within the GST family (involved in xenobiotic degradation and metabolism) was identified and upregulated, consistent with the increased GST activity observed by Chen et al. in tobacco cutworm larvae after Pb treatment.

## 4. Conclusions

In summary, in this study, we analyzed the midgut transcriptome of silkworms fed with mulberry leaves soaked in C_4_H_6_O_4_Pb·3(H2O) and ddH2O. A total of 11,567 unigenes were identified and annotated using functional databases such as NR, Swiss-Prot, Pfam, eggNOG, GO, and KEGG. Among the identified 1265 DEGs, 907 were upregulated, and 358 were downregulated. This study elucidated the transcriptome of silkworms and deepened our understanding of detoxification-related genes in silkworms. It also provides a new perspective for understanding the detoxification defense mechanisms of silkworms against Pb exposure, aiding further research.

## Data Availability

The original transcriptome data has been submitted to the NCBI database with the accession number PRJNA1111735.

## Supplementary Materials

The following supporting information can be downloaded at: https://www.mdpi.com/article/10.3390/ani14121822/s1, Table S1. Primer sequences for qRT-PCR, Table S2. RNA-seq reads, Table S3. Summary of the assembly of *B. mori*, Table S4. A summary of unigenes was annotated in databases.
