# Supplementary material for: Transcriptome Analysis Reveals Antioxidant Defense Mechanisms in the Silkworm Bombyx mori after Exposure to Lead
Source: Animals (Basel). 2024 Jun 19;14(12):1822. doi: 10.3390/ani14121822 (PMC11201215; doi:10.3390/ani14121822)
Supplement: Supplementary file 1 [file animals-14-01822-s001.zip › animals-2993979-supplementary.pdf]

Table S1. Primer sequences for qRT-PCR.

Gene name	Primer sequence (5' to 3')	Product length (bp)
Actin	F: CGGCTACTCGTTCACTACC	147
	R: CCGTCGGGAAGTTCGTAAG	
CYP49A1	F: AATCAGGAGCCGACTTGTCCC	157
	R: AAGGCATCGTTGAATGTTTGG	
HSP90B1	F: CACTGAAATGCCGAATAGTCTAAC	228
	R: GACCTCTTCCACTGTGCCAAC	
Txn2	F: ACTACTTACTCCTCGCCTTGA	147
	R: TTCCCATTCTTTATCGCTACT	
CuZn-SOD	F: TCGTGGTGATGTTAGCGGAACT	148
	R: CGACATGGCGTACAGCAGAACT	
CYP4C1	F: CTGTCTAACGAAAGGGATG	121
	R: CTGTCTAACGAAAGGGATG	
CYP4G15	F: CTGTCTAACGAAAGGGATG	251
	R: CTGTCTAACGAAAGGGATG	
CYP6AB4	F: CTTCACGCTCCATCAACT	184
	R: GCACTCTCCAAGTATCTCAT	
GST03	F: TTGAGGTGGAAGATGGAGTATGT	260
	R: TGTGCCACGATTCCTTAACTG	
HSP70	F: CTGTGAAGAATGAGGGTG	202
	R: CTGATACTTTGGCAATAGA	

Gene name	Primer sequence (5' to 3')	Product length (bp)
CYP6K1	F: GTTTGCCGGACTTGATACAAC	243
	R: CTTACTACGAACGGGAGGGAC	
CYP6B6	F: CATTGTCGCTCTTGTTTATTTG	148

	R: ATCCACAGCAGTTTGGGTCAT	
HSP90	F: ATCCACAGCAGTTTGGGTCAT	259
	R: CGAACGGCGACTTCTCCAC	

Table S2. RNA-seq reads.

Sample	Raw reads	Raw bases	Clean reads	Clean bases	Error rate(%)	Q20(%)	Q30(%)	GC content(%)	Total reads	Total mapped	Multiple mapped	Uniquely mapped
G48hPb	43522228	6.572E+09	41385924	6.128E+09	0.0255	97.76	93.78	50.92	41385924	37822014(91.39%)	3229608(7.8%)	34592406(83.58%)
A48hCK	54308482	8.201E+09	51994280	7.679E+09	0.0256	97.72	93.72	51.78	51994280	48004519(92.33%)	5355196(10.3%)	42649323(82.03%)

Table S3. Summary of the assembly of *B. mori*.

Length	Number
0~200	587
201~400	2608
401~600	2008
601~800	1712
801~1000	1528
1001~1200	1324
1201~1400	1263
1401~1600	1187
1601~1800	1024
>1800	8666
total	21907

Table S4. A summary of unigenes was annotated in databases.

	Expre_Gene number (percent)	Expre_Transcript number (percent)	All_Gene number (percent)	All_Transcript number (percent)
GO	8,255 (0.8258)	7,632 (0.8243)	11,681 (0.7269)	11,681 (0.7269)
KEGG	6,065 (0.6067)	5,615 (0.6064)	8,036 (0.5001)	8,036 (0.5001)
EggNOG	9,644 (0.9648)	8,938 (0.9653)	14,389 (0.8955)	14,389 (0.8955)
NR	9,884 (0.9888)	9,163 (0.9896)	15,088 (0.939)	15,088 (0.939)
Swiss-Prot	7,640 (0.7643)	7,069 (0.7635)	9,796 (0.6096)	9,796 (0.6096)
Pfam	7,806 (0.7809)	7,223 (0.7801)	10,330 (0.6429)	10,330 (0.6429)
Total_anno	9,927 (0.9931)	9,204 (0.9941)	15,308 (0.9526)	15,308 (0.9526)
Total	9,996(1.0)	9,259 (1.0)	16,069 (1)	16,069 (1)
